# Remnant-Like Particle Cholesterol and the Risk of Major Adverse Cardiovascular Events: A Systematic Review and Meta-Analysis

**DOI:** 10.3390/jcdd9120452

**Published:** 2022-12-11

**Authors:** Jie Yang, Yuangengshuo Wang, Ziwei Xi, Yue Ma, Chunli Shao, Wenyao Wang, Yi-Da Tang

**Affiliations:** 1 Department of Cardiology, State Key Laboratory of Cardiovascular Disease, Fuwai Hospital, National Center for Cardiovascular Diseases, Chinese Academy of Medical Sciences and Peking Union Medical College, Beijing 100037, China; 2 Department of Cardiology and Institute of Vascular Medicine, Peking University Third Hospital, Key Laboratory of Molecular Cardiovascular Science, Ministry of Education, 49 Huayuanbei Road, Beijing 100191, China

**Keywords:** remnant-like particle cholesterol, coronary artery disease, residual cardiovascular risk

## Abstract

Background: The remnant-like particle cholesterol (RLP-C) has been demonstrated to be associated with residual cardiovascular risk. The meta-analysis aimed to evaluate the impact of baseline RLP-C on the incidence of major cardiovascular adverse events (MACEs) in patients with coronary artery disease (CAD). Methods: A systematic literature search was performed in PubMed and Embase electronic databases from the inception of the databases through 1 October 2022. Studies evaluating the association between baseline RLP-C and the risk of MACEs in patients with CAD were included. Hazard ratios (HRs) with 95% confidence intervals (CIs) were pooled by a random-effect method (RLP-C analyzed as a categorical variable) and a fixed-effects model (RLP-C analyzed as a continuous variable). Results: Ten studies including 18,053 subjects were finally included in this meta-analysis. In our pooled analysis, compared to CAD patients with the lowest RLP-C category, the CAD patients with the highest RLP-C category had a significantly higher risk of future MACEs during follow-up (HR 1.79, 95% CI, 1.42–2.26, I^2^ = 60.31%, *p* < 0.01), which was consistent with outcomes of meta-analysis with the RLP-C analyzed as a continuous variable (HR 1.40, 95% CI, 1.28–1.53, I^2^ = 38.20%, *p* < 0.01). The sensitivity analysis confirmed the robustness of the results, and no significant publication bias was identified. Conclusion: The present meta-analysis suggests that the RLP-C was associated with an increased risk of long-term MACEs in patients with CAD at baseline. It is necessary to conduct randomized controlled trials to explore whether reducing the RLP-C level is conducive to reducing residual cardiovascular risk, even coronary plaque regression.

## 1. Introduction

Coronary artery disease (CAD) is the most common cause of death all over the world, resulting in a huge economic and medical burden [1,2]. The rise of cholesterol is one of the widely accepted mechanisms that cause atherosclerosis, and the oxidized low-density lipoprotein (LDL) particles play an important role [3,4]. Therefore, lipid lowering therapy focusing on lowering low-density lipoprotein cholesterol (LDL-C) has been widely applied to the primary and secondary prevention of CAD [5]. With the application of statins, ezetimibe, and proprotein convertase subtilisin/kexin type 9 (PCSK9) inhibitors, the LDL-C level and the risk of adverse cardiovascular events of CAD patients have been significantly decreased [6,7,8]. However, there is also a considerable residual cardiovascular risk that may be driven by non-high density lipoprotein cholesterol (non-HDL-C), lipoprotein (a), and remnant-like particle cholesterol (RLP-C) [9,10,11].

In recent years, triglyceride-rich lipoproteins (TRLs) including chylomicron remnant, intermediate-density lipoprotein (IDL), and very low-density lipoprotein (VLDL), have been regarded as potential cardiovascular risk factors besides LDL-C, especially for patients with metabolic disorders [12,13]. Most studies have suggested that the RLP-C, as the cholesterol content of a subset of the TRLs, was significantly associated with the increasing risk of CAD and major cardiovascular adverse events (MACEs) [14,15,16]. However, a few studies indicated that there was no significant association between RLP-C and cardiovascular events [17,18]. In view of the conflicting data, we carried out a systematic review and meta-analysis to explore the association between RLP-C and the risk of MACEs in patients with CAD, and to obtain a quantitative estimate of the risk.

## 2. Method

### 2.1. Literature Retrieval Strategy

We searched published studies in PubMed and Embase electronic databases from the inception of these databases to 1 October 2022, using the following retrieval methods described in Item S1 in Supplementary Materials. Only full-length articles were screened, while conference abstracts would be excluded. There was no language limitation.

### 2.2. Inclusion and Exclusion Criteria

Inclusion criteria: (1) cohort, or nest case-control study evaluating the association between RLP-C and the risk of MACEs in CAD patients, (2) RLP-C was measured at baseline, (3) the endpoints of studies included MACEs, (4) studies reported hazard ratio (HR), relative risk (RR), or Odds ratio (OR) with 95% confidence intervals (CI) after adjustment of confounding factors, (5) the median follow-up time was over six months.

Exclusion criteria: (1) not all participants were diagnosed with CAD at baseline, (2) cross-sectional studies, and (3) conference abstract without full text. The screening of eligible studies was conducted by Yang and Wang, and disagreements were judged by a third reviewer (Y.-D.T.)

RLP-C was defined as the cholesterol content of a subset of TRLs, i.e., chylomicron remnants, VLDL, and IDL in the non-fasting state, and VLDL and IDL in the fasting state; calculated by the formula: TC minus HDL-C minus LDL-C. Non-HDL-C was calculated by the formula: TC minus HDL-C. The definition of MACEs was in accordance with the criteria of the original research. The MACEs were typically defined as a composite endpoint including cardiac death, myocardial infarction, stroke and repeat revascularization. When multiple studies deriving from the same data source were appropriate, we incorporated only the one with the maximum sample size. Moreover, if both the outcomes of calculated RLP-C and measured RLP-C were available, we used the outcomes of calculated RLP-C. We used the Newcastle-Ottawa quality assessment scale to evaluate the quality of cohort and nest case-control studies [19], where 7–9 points was assumed high quality, 5–6 points was assumed moderate quality, and 0–4 points was assumed low quality.

### 2.3. Data Extraction

Two reviewers (Yang and Wang) extracted detailed data from included studies. The extracted data included the last name of the first author, year of publication, country of origin, study design, sample size, subject characteristics (age, sex, type of CAD and mean LDL-C), follow-up time, outcomes reported, and confounding factors adjusted in the multivariate regression analyses.

### 2.4. Statistical Analysis

STATA version 17.0 was used to perform statistical analysis. The association of baseline RLP-C with the risk of future MACEs was evaluated by calculating the pooled HR and 95% CI. We extracted the HR and 95% CI of the future MACEs in subjects with the highest RLP-C group compared with those with the lowest RLP-C group, when the studies analyzed RLP-C as a categorical variable. When the studies analyzed RLP-C as a continuous variable, we extracted the HR and 95% CI of future MACEs per 1-SD/1-log unit increment of the RLP-C according to the reporting of the original studies. We used the Galbraith plots, the I^2^ statistic and Cochran’s Q test to assess the between-study heterogeneity. When the *p* value of the Q test was <0.1 and I^2^ was >50%, we used the random-effects model to calculate the pooled HR and 95% CI, otherwise, we used the fixed-effects model to calculate them. The significance of the pooled HR was assessed by the Z test (*p* < 0.05 was regarded as statistically significant). Subgroup analysis was performed to explore the potential source of heterogeneity. Moreover, sensitivity analysis (excluding one study at a time) was carried out to assess the robustness of the outcomes. Moreover, we would conduct funnel plots to evaluate potential publication bias.

## 3. Results

### 3.1. Characteristics of Included Studies

**Figure 1:**
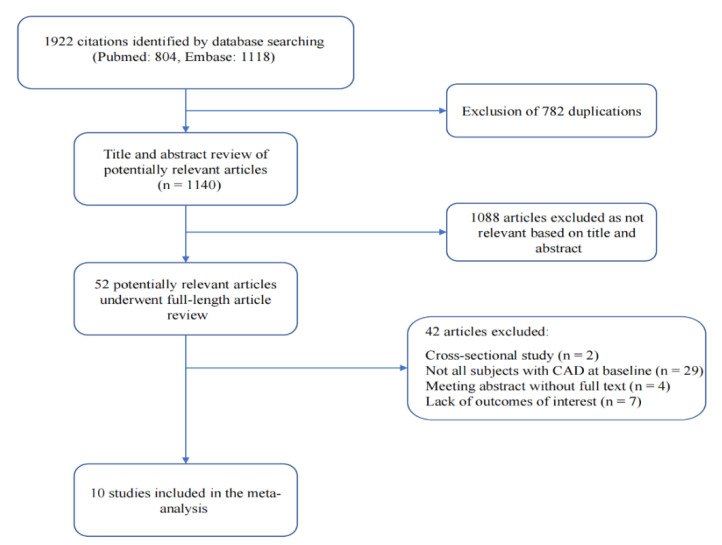
Flow chart of meta-analysis for exclusion/inclusion of individual articles.

The present meta-analysis finally included 10 studies comprising 18,053 subjects according to our retrieving methods and screen criteria (Figure 1). These studies included participants from two countries (four from Japan, six from China). Most European and American studies were excluded because they included the general population rather than the population with coronary heart disease at baseline. Half of the studies were prospective cohort studies, and half of the studies were retrospective cohort studies. Four studies included subjects with acute coronary syndrome (ACS), four studies included patients with stable CAD, and two studies included patients with CAD. The average age of the included patients ranged from 55 to 70 years old, and the proportion of female patients varied from 9 to 43%. The median follow-up time ranged from 12 to 54.9 months. All the studies scored more than 7 points based on the Newcastle–Ottawa quality assessment scale, suggesting good study quality (Table 1).

### 3.2. Meta-Analysis Results

**Figure 2:**
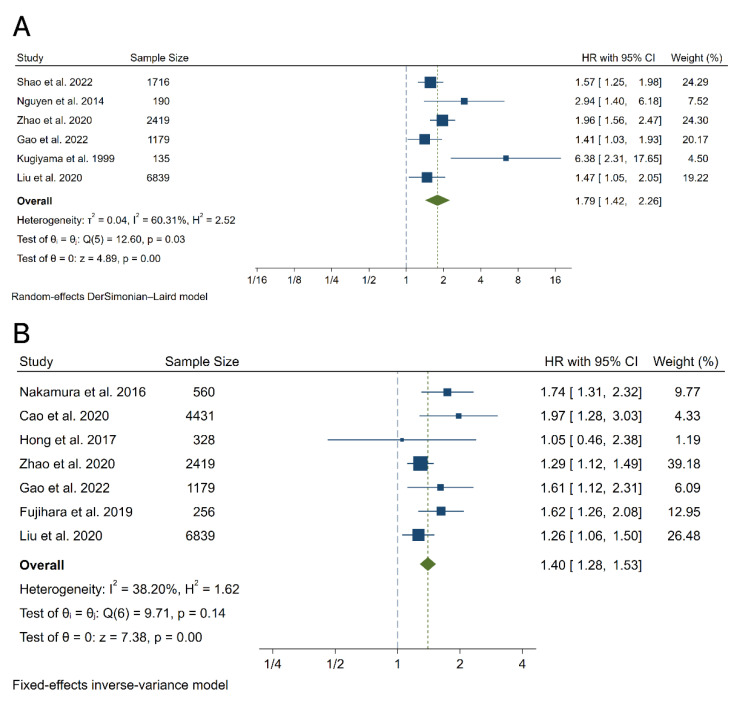
Forest plots for the meta-analysis of the association between the RLP-C and the risk of MACEs. (**A**) Meta-analysis with the RLP-C analyzed as a categorical variable. (**B**) Meta-analysis with the RLP-C analyzed as a continuous variable. References involved in the study: [14,15,17,20,22,23,24,25,26].

**Figure 3:**
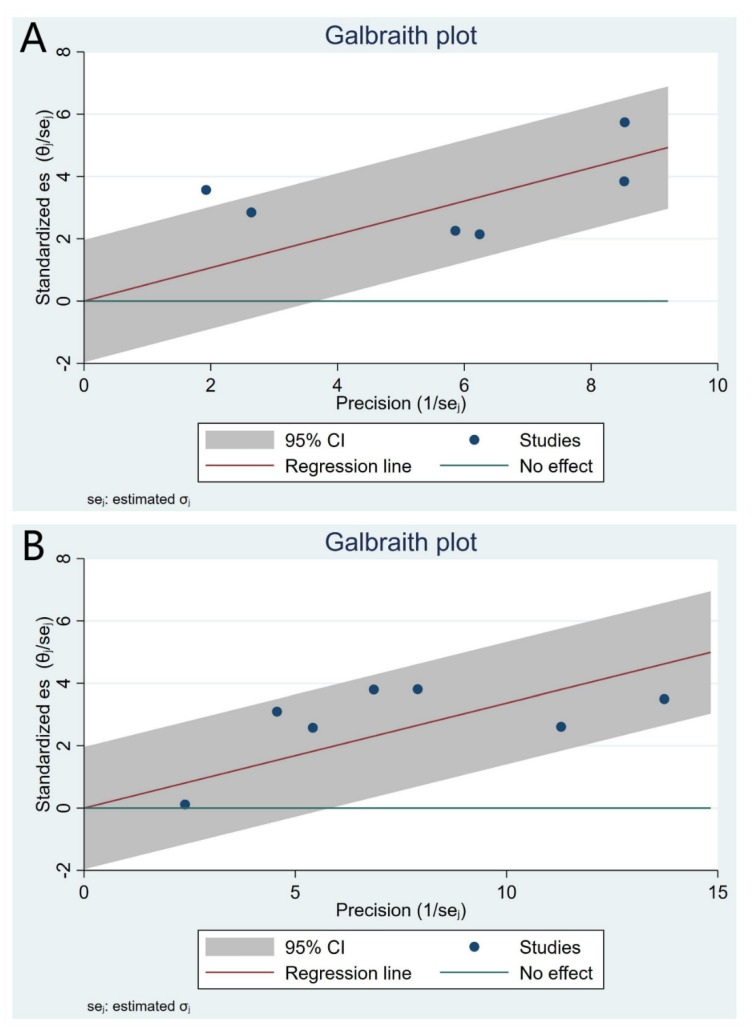
Galbraith plots for heterogeneity test of the meta-analysis. (**A**) The RLP-C analyzed as a categorical variable. (**B**) The RLP-C analyzed as a continuous variable.

**Figure 4:**
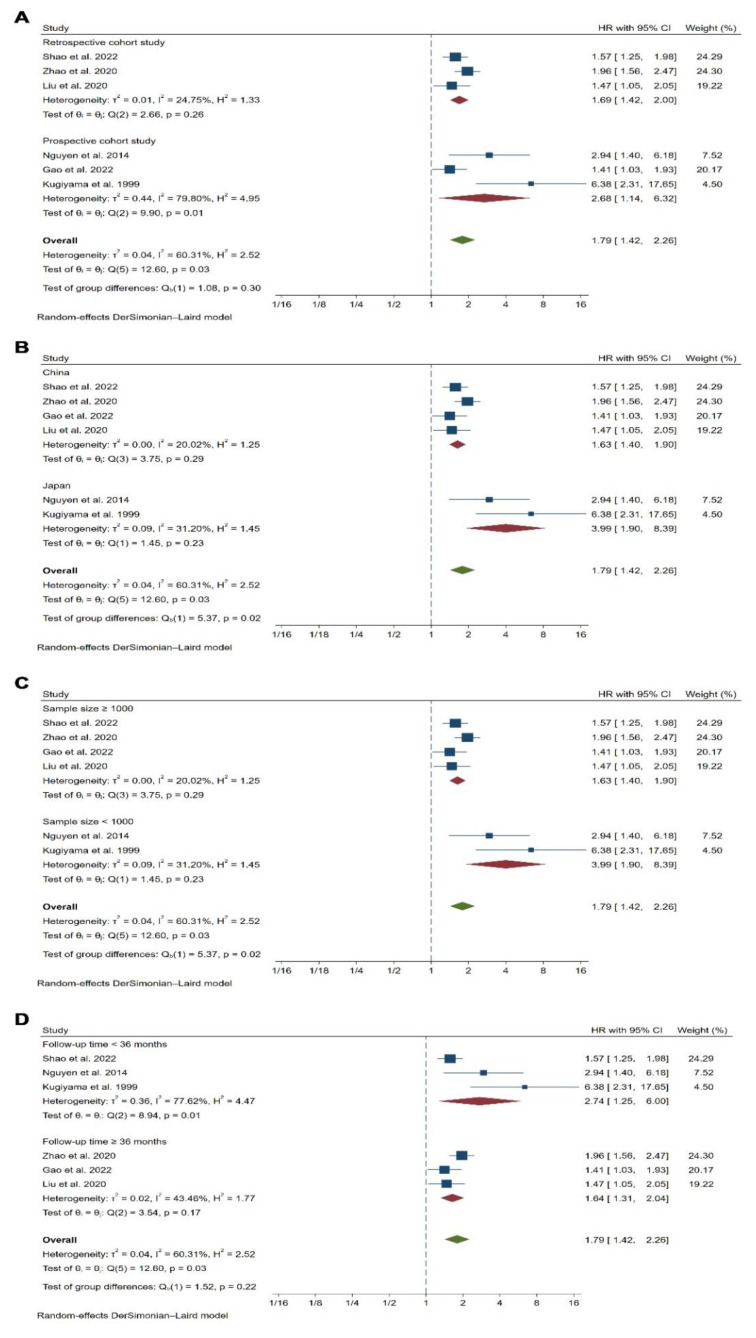
Subgroup analyses for the association between the RLP-C analyzed as a categorical variable and the risk of MACEs. (**A**) Subgroup analysis according to the study design. (**B**) Subgroup analysis according to the country of origin. (**C**) Subgroup analysis according to the sample size. (**D**) Subgroup analysis according to the follow-up time. References involved in the study: [14,15,20, 22,24,26].

In our pooled analysis, compared to CAD patients with the lowest RLP-C category, the CAD patients with the highest RLP-C category had a significantly higher risk of future MACEs during follow-up (HR 1.79, 95% CI, 1.42–2.26, *p* < 0.01) with moderate heterogeneity between studies (I^2^ = 60.31%, Q = 12.60, *p* = 0.03) (Figure 2A and Figure 3A), which was consistent with outcomes of meta-analysis with the RLP-C analyzed as a continuous variable (HR 1.40, 95% CI, 1.28–1.53, *p* < 0.01, I^2^ = 38.20%, Q = 9.71, *p* = 0.14) (Figure 2B and Figure 3B). To explore the potential source of heterogeneity of the meta-analysis outcomes with the RLP-C analyzed as a categorical variable, we conducted subgroup analysis according to study design, country of origin, sample size, and follow-up time. The results showed that the sample size and the country of origin might contribute to the heterogeneity (Figure 4). Moreover, we conducted sensitivity analysis by excluding one study at a time, and the result indicated that no specific study affected the primary outcomes significantly (Supplementary Figure S1). The funnel plots were almost symmetric visually, indicating a possible low risk of publication bias (Supplementary Figure S2). Moreover, the Begg’s test and the Egger’s test were unable to be conducted because the available studies were not more than ten.

## 4. Discussion

The present meta-analysis supplied a systematic review of published articles and a quantitative estimation of the association between the RLP-C and the risk of MACEs in patients with CAD. The results suggested that the CAD patients with the higher baseline RLP-C level had a significantly higher risk of future MACEs during follow-up regardless of whether the RLP-C was regarded as a category variable or continuous variable, with low risk of publication bias. Several studies were used twice because they reported results regarding RLP-C as categorical variable and continuous variable, respectively. The outcomes of subgroup analysis indicated that the moderate heterogeneity might result from different sample size and country of origin. Moreover, the result of sensitivity analysis suggested the stability of our primary outcomes. In view of the results above, the RLP-C may be the next therapeutic target for patients with a considerable cardiovascular residual risk.

To our knowledge, the present study is the first meta-analysis estimating the association between the baseline RLP-C and future MACEs in patients diagnosed with CAD at baseline. As we all know, the LDL-C-lowering therapy is the primary treatment for patients with CAD. Nevertheless, some patients are still under considerable cardiovascular residual risk after effective LDL-C-lowering therapy. In that case, the TRLs may play an important role in the cardiovascular residual risk, especially for patients with metabolic disorders, such as diabetes mellitus, metabolic syndrome, and insulin resistance [13,27]. In recent years, the RLP-C, as the cholesterol content of a subset of the TRLs, has been emphasized in the primary prevention and secondary prevention of CAD. Quispe et al. [28] included 17,532 ASCVD-free subjects at baseline from three prospective cohorts. The result of pooled analysis showed that log RLP-C correlated with increasing ASCVD risk (HR, 1.65, 95% CI, 1.45–1.89) after median follow-up of 18.7 years. Shao et al. [15] consecutively included 1716 ACS patients undergoing percutaneous coronary intervention. They defined the primary endpoint as MACEs, including all-cause mortality, non-fatal stroke, non-fatal myocardial infarction, and unplanned repeat revascularization, and the median follow-up time was 2.5 years. The outcomes suggested that baseline RLP-C > 75th was associated with the higher risk of long-term MACEs (HR, 1.572, 95%CI, 1.25–1.98, *p* < 0.001). Fujihara and his colleagues [25] consecutively enrolled 247 individuals with stable CAD and LDL-C levels lower than 70 mg/dL on statin treatment. During a mean follow-up time of 3.2 years, 33 MACEs occurred. The outcomes of multivariate Cox regression analysis indicated that RLP-C was associated with increasing risk of adverse cardiovascular events in patients with stable CAD (HR1.62, 95% CI, 1.26–2.07, *p* < 0.01).

The potential mechanism of the correlation between elevated RLP-C and the risk of MACEs in patients with CAD may include the following aspects. First, the RLP-C can invade the arterial intima and can be absorbed directly by macrophages, promoting the formation of foam cells and accelerating the progression of atherosclerosis, while the LDL can only be taken up by macrophages after being oxidative modified [27,29]. Second, the RLP-C is closely related to low-grade inflammation, with a 1-mmol/L increasing remnant cholesterol associated causally with a 28% increasing C-reactive protein [30]. Third, previous studies suggested the RLP-C was associated with endothelial dysfunction by impairing acetylcholine-induced vasodilatation and flow-mediated endothelium-dependent dilatation [31]. Fourth, the RLP-C promote the expression of plasminogen activator inhibitor-1 and induce platelet aggregation, increasing the risk of thrombus [32,33]. The atherogenic, pro-inflammatory and thrombogenic role of the RLP-C may be the underlying mechanism of the correlation between the RLP-C and cardiovascular events.

Under the condition that LDL-C lowering therapy was widely used, the inflammation and other lipid indicators, including non-HDL-C, TG, LP (a), RLP-C and Apo B, may play important roles in the residual cardiovascular risk. The cholesterol component of TRLs is regarded as the causal culprit instead of TG, because Chylomicron is unable to enter the endarterium and TG can be disintegrated by most cells [34]. Moreover, Quispe et al. [28] demonstrated that the RLP-C was associated with residual cardiovascular risk beyond Apo B, LDL-C, and non-HDL-C. Therefore, the residual inflammation risk and residual lipid (RLP-C and LP (a)) risk should be emphasized. It is the time to carried out randomized controlled trials to explore whether reducing the RLP-C level is conducive to reducing residual cardiovascular risk, even coronary plaque regression.

The study has some limitations. First, all studies included in the meta-analysis were from Asia, making it difficult to extrapolate the results to other races. Second, the type of CAD, the definitions of MACEs, follow-up time, and confounding factors adjusted were different across the studies, which may lead to potential heterogeneity. Third, as one of the sources of residual cardiovascular risk, LP(a) was adjusted as a covariate in only two studies, which may obscure the true effect of RLP-C. Fourth, the available studies were limited, and the sample sizes of studies varied greatly. The Begg’s test and the Egger’s test were unable to be conducted because the available studies were not more than ten. The results of subgroup analysis indicated that the sample size might contribute to the moderate heterogeneity. Finally, it remains unclear whether the association between the RLP-C and the risk of MACEs is linear or non-linear.

## 5. Conclusions

The present meta-analysis suggests that the RLP-C was associated with an increased risk of long-term MACEs in patients with CAD at baseline. It is necessary to conduct randomized controlled trials to explore whether reducing the RLP-C level is conducive to reducing residual cardiovascular risk, even coronary plaque regression.

## Figures and Tables

**Table 1:** Main characteristics of the studies included in this meta-analysis of the association between the RLP-C and MACEs.

	Country	Study Design	Subjects	Sample Size	Sex Female	Age (Years)	RLP-C Analysis	Mean LDL-C (mmol/L)	Follow-Up Time (Months)	Outcome Reported	Confounder Adjustment	Quality Assessment (Newcastle–Ottawa Scale)
Shao et al. 2022 [15]	China	Retrospective cohort study	Patients with ACS undergoing PCI	1716	23.3%	60 ± 10	RLP-C > 75th vs. RLP-C ≤ 75th	2.44 ± 0.80	30.9	MACE (354)	Age, sex, BMI, current smoking, hypertension, diabetes, past MI, past PCI, CKD, statins on admission, discharged drugs, complete revascularization, STEMI, hs-CRP, GRACE risk score, left main or multivessel disease.	Selection: 4 Comparability: 2 Outcome: 2
Nguyen et al. 2014 [20]	Japan	Prospective cohort study	Patients with ACS undergoing PCI	190	27.9%	70.2 (63.0 − 79.0)	RLP-C ≥ 5.4 mg/dL vs. RLP-C < 5.4 mg/dL	2.57 ± 0.80	30	MACE (42), Cardiac death (2), MI (10), Ischemia-driven revascularization (25), Stroke (30)	Age, sex, smoking, BMI, DM, HTN, Multivessel CAD, hs-CRP, HbA1c, TG, HDL-C, and LDL-C.	Selection: 4 Comparability: 2 Outcome: 2
Nakamura et al. 2016 [21]	Japan	Prospective cohort study	Patients with stable CAD	560	43.0%	64 ± 9	Continuous	2.31 (1.92–2.51)	33	MACE (40), Cardiac death (13), MI (2), Ischemia-driven revascularization (17), Stroke (8)	Multivessel CAD, CRP, eGFR, BNP, non-HDL-C, and ApoA-I.	Selection: 4 Comparability: 1 Outcome: 2
Cao et al. 2020 [22]	China	Retrospective cohort study	Patients with CAD	4431	28.9%	58.32 ± 12.29	Continuous	2.44 ± 0.89	61.2	MACE (541), Cardiac death (75), UAP requiring hospitalization (132), MI (44), Repeat revascularization (181), Stroke (109)	Age, sex, smoking, BMI, DM, HTN, Family history of CAD, Baseline statin, TC, TG, HDL-C, non-HDL-C, Apo B, and LDL-C.	Selection: 4 Comparability: 2 Outcome: 2
Hong et al. 2017 [17]	China	Retrospective Cohort study	Patients with stable CAD and diabetes mellitus	328	36.2%	59.2 ± 9.4	Continuous	2.50 ± 1.00	12	MACE (47), Cardiac death (3), UAP requiring hospitalization (5), MI (8), Repeat revascularization (32)	Age, sex, smoking, BMI, HTN, Family history of CAD, Gensini scores, Lp (a), HbA1c, hs-CRP, Fibrinogen, Neutrophil count and LDL-C.	Selection: 4 Comparability: 2 Outcome: 1
Zhao et al. 2020 [14]	China	Retrospective cohort study	Patients with NSTE-ACS undergoing PCI	2419	28.2%	60.08 ± 8.97	RLP-C > 50th vs. RLP-C ≤ 50th, Continuous	2.50 ± 0.88	36	MACE (454), all-cause death (21), MI (117), ischemia-driven revascularization (316)	Age, BMI, heart rate, SBP, DM, prior MI, prior PCI, prior CABG, prior stroke, TG, TC, HDL-C, hs-CRP, eGFR, FBG, HbA1c, LVEF, principal diagnosis, discharged drugs, Left main disease, muti-vessel disease, CTO disease, diffuse disease, bifurcation disease, and number of stents.	Selection: 4 Comparability: 2 Outcome: 1
Gao et al. 2022 [23]	China	Prospective cohort study	Patients with MINOCA	1179	36.5%	55.70 ± 11.8	RLP-C > 50th vs. RLP-C ≤ 50th, Continuous	2.29 ± 0.76	41.7	MACE (168), All-cause death (18), UAP or HF requiring hospitalization (119), MI (41), Repeat revascularization (46), Stroke (109)	Age, sex, BMI, MI type, HTN, DM, and dyslipidemia.	Selection: 4 Comparability: 2 Outcome: 2
Kugiyama et al. 1999 [24]	Japan	Prospective cohort study	Patients with CAD	135	34.0%	65.00 ± 9.70	T3 vs. T1-T2	NA	26.8	MACE (45)	Age, sex, smoking, HTN, DM, hypercholesterolemia, hypertriglyceridemia, low levels of HDL cholesterol, stenosis of left main coronary artery, and the number of diseased coronary arteries.	Selection: 4 Comparability: 2 Outcome: 1
Fujihara et al. 2019 [25]	Japan	Prospective cohort study	Patients with stable CAD	256	9.0%	67.0 (60.0 –74.0)	Continuous	1.60 (1.45–1.73)	38	MACE (33), Cardiac death (2), HF requiring hospitalization (9), MI (1), ischemia-driven revascularization (13), Stroke (30), PAD requiring endovascular treatment (1), aortic aneurysms requiring surgical treatment (3)	Smoking, TG, Lp (a), HbA1c, and ApoB.	Selection: 4 Comparability: 1 Outcome: 2
Liu et al. 2020 [26]	China	Retrospective cohort study	Patients with stable CAD	6839	27.6%	58.10 ± 10.70	RLP-C > 50th vs. RLP-C ≤ 50th, Continuous	2.44 ± 0.92	54.9	MACE (462), Cardiac death (197), MI (94), Stroke (171)	Age, sex, smoking status, prior MI, HTN, DM, LVEF, TG, LDL-C, HDL-C creatinine, statin use and types at admission, and statintypes on discharge.	Selection: 4 Comparability: 2 Outcome: 2

Abbreviation: RLP-C, remnant-like particle cholesterol; MACEs, major cardiovascular adverse events; CAD, coronary artery disease, ACS, acute coronary syndrome; PCI, percutaneous coronary intervention; BMI, body mass index; MI, myocardial infarction; STEMI, ST-segment elevated myocardial infarction; CKD, chronic kidney disease; hs-CRP, high-sensitivity C-reactive protein; GRACE, Global Registry of Acute Coronary events; DM, diabetes mellitus; HTN, hypertension; TC, total cholesterol; TG, triglyceride; HDL-C, low-density lipoprotein cholesterol; LDL-C, low-density lipoprotein cholesterol; eGFR, estimated glomerular filtration rate; Apo A-I, BNP, B-type natriuretic peptide; apolipoprotein A-I; Apo B, apolipoprotein B; Lp (a), lipoprotein a; CABG, coronary artery bypass graft; FBG, fasting blood glucose; LVEF, left ventricular ejection fraction; CTO, chronic total occlusion; UA, uric acid; ALT, alanine aminotransferase; Cre, creatinine; CT, computed tomography; DM, diabetes mellitus; HTN, hypertension; CVD, cardiovascular disease; TC, total.cholesterol.

## Data Availability

All data and materials used in this research are freely available in electronic databases (PubMed: Embase). References have been provided.

## Supplementary Materials

The following supporting information can be downloaded at: https://www.mdpi.com/article/10.3390/jcdd9120452/s1, Item S1: Search Strategy; Supplementary Figure S1. Sensitivity analysis for leave one study out. A The RLP-C analyzed as a categorical variable. B The RLP-C analyzed as a continuous variable; Supplementary Figure S2. Funnel plots for publication bias test of the meta-analysis. A The RLP-C analyzed as a categorical variable. B The RLP-C analyzed as a continuous variable.
