# Supplementary material for: Remnant-Like Particle Cholesterol and the Risk of Major Adverse Cardiovascular Events: A Systematic Review and Meta-Analysis
Source: J Cardiovasc Dev Dis. 2022 Dec 11;9(12):452. doi: 10.3390/jcdd9120452 (PMC9781984; doi:10.3390/jcdd9120452)
Supplement: Supplementary file 1 [file jcdd-09-00452-s001.zip › jcdd-2091582-supplementary.pdf]

Item S1: Search Strategy

PubMed:

("remnant-like particle cholesterol" [Supplementary Concept] OR "remnant cholesterol" OR "remnant lipoprotein" OR "RLP-C")

Embase:

('remnant like particle cholesterol'/exp OR 'remnant cholesterol' OR 'remnant lipoprotein' OR 'RLP-C')

Supplementary Figure S1

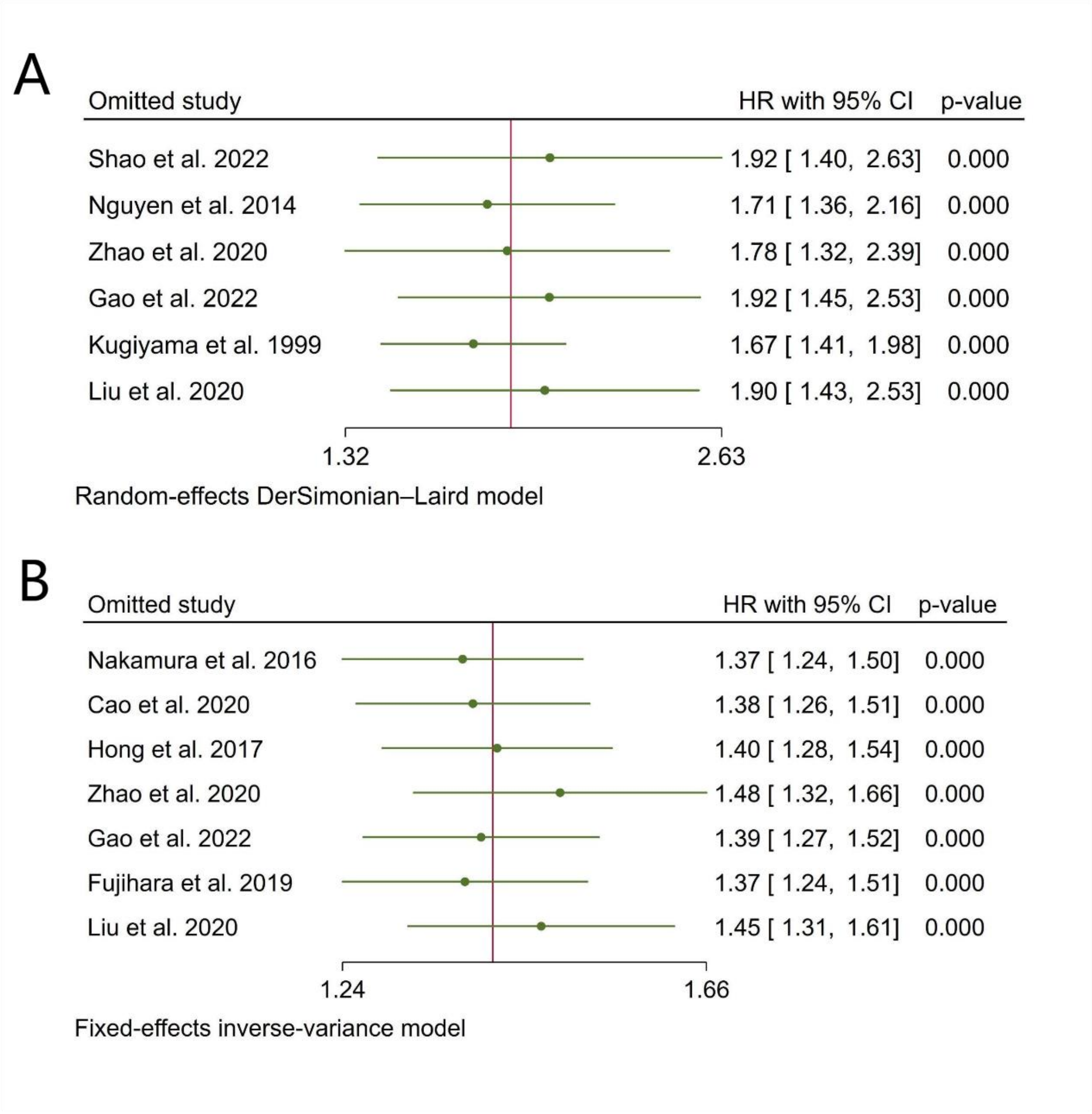

Supplementary Figure S1. Sensitivity analysis for leave one study out. **A** The RLP-C analyzed as a categorical variable. **B** The RLP-C analyzed as a continuous variable. References involved in the study: [14,15,17,20,22–26]

Supplementary Figure S2

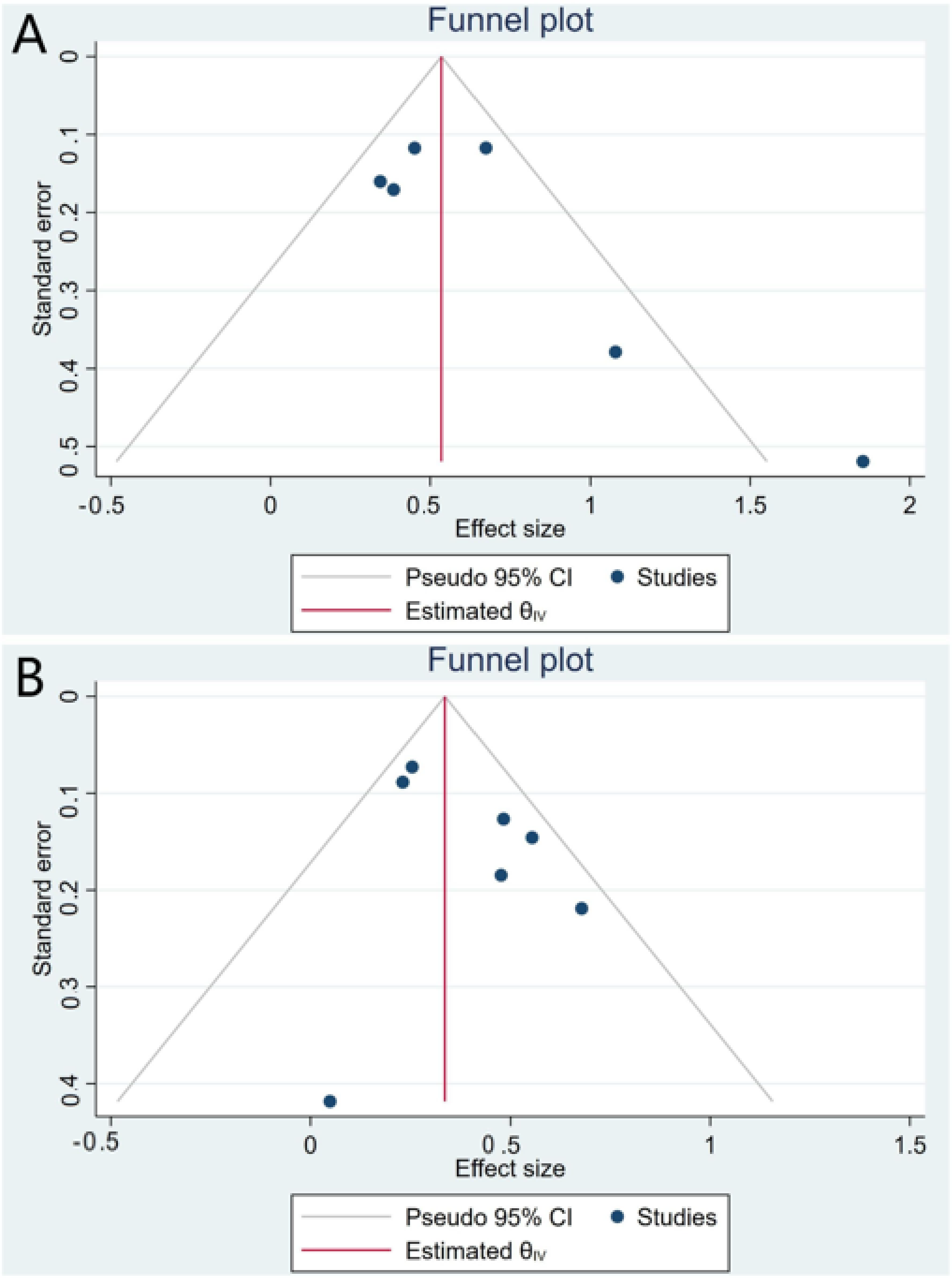

Supplementary Figure S2. Funnel plots for publication bias test of the meta-analysis. **A** The RLP-C analyzed as a categorical variable. **B** The RLP-C analyzed as a continuous variable
